# Reliability and Validity of the Simplified Chinese Version of the Aberrant Behavior Checklist in Chinese Autism Population

**DOI:** 10.3389/fpsyt.2020.545445

**Published:** 2020-10-14

**Authors:** Siuching Kat, Lingzi Xu, Yanqing Guo, Junhong Ma, Zenghui Ma, Xinzhou Tang, Yulu Yang, Hui Wang, Xue Li, Jing Liu

**Affiliations:** ^1^Institute of Mental Health, Peking University Sixth Hospital, Beijing, China; ^2^National Clinical Research Center for Mental Health Disorders and Key Laboratory of Mental Health, Ministry of Health, Peking University, Beijing, China; ^3^Education Department, Peking University Health Science Center, Beijing, China

**Keywords:** aberrant behavior checklist, autism, reliability, validity, Chinese population

## Abstract

**Background:** The Aberrant Behavior Checklist (ABC) is a widely used scale in autism clinical intervention research for the assessment of core symptoms and comorbid emotional and behavioral problems among people with autism. The aim of this study was to examine the psychometric properties of the Simplified Chinese version of the Aberrant Behavior Checklist (SC-ABC) using a sample of people with autism in a Chinese population.

**Methods:** In total, we enrolled 799 patients aged 1.5–33 years old. We collected data using the SC-ABC (*n* = 799), Autism Behavior Checklist (*n* = 743), Attention Deficit Hyperactivity Disorder Rating Scale-IV (ADHD-RS-IV) (*n* = 433) and Achenbach Child Behavior Checklist (CBCL) (*n* = 319). Eighty-four patients were separately assessed with the SC-ABC by two caregivers simultaneously. Forty-four patients were assessed with the SC-ABC again by same caregiver 2 weeks after the first assessment. SC-ABC data from the whole sample were used for confirmatory factor analysis. We evaluated criterion validity using Spearman's correlation coefficient between scores of the SC-ABC and scores of the Autism Behavior Checklist, ADHD-RS-IV and CBCL separately in the whole sample and different age groups. We calculated the intragroup correlation coefficients and Spearman's correlation coefficient for interrater reliability in 84 samples and test-retest reliability in 44 samples. We conducted Cronbach's α for internal consistency.

**Results:** For the SC-ABC, the intragroup correlation coefficients of five subscales and the total score in interrater and test-retest reliability ranged from 0.87 to 0.92 and from 0.93 to 0.97 (all *P* < 0.01). The Spearman's correlation coefficient of five subscales and the total score in interrater and test-retest reliability ranged from 0.78 to 0.85 and 0.86 to 0.94, respectively (all *P* < 0.01). Cronbach's α of five subscales and the total score ranged from 0.75 to 0.96 (all *P* < 0.01). The Spearman's correlation coefficient for criterion validity for the whole sample and different age groups ranged from 0.39 to 0.76 (all *P* < 0.01). The model fit for the original five factor model was acceptable, with fit indices of SMR = 0.062 and RMSEA = 0.052.

**Conclusions:** The SC-ABC has satisfactory psychometric properties and can be used in the assessment of core symptoms and comorbid emotional and behavioral problems in patients with autism.

## Introduction

Autism is a neurodevelopmental disorder with core symptoms of abnormal functioning in social interaction and communication, and restricted, repetitive behavior (1). It is often comorbid with emotional or behavioral problems or mental disorders. World Health Organization estimated the global prevalence of autism spectrum disorder (ASD) to be ~1% (2), which was similar to that in China reported by Xiang Sun et al. in 2019 (3). ASD is associated with functional impairment of affected individuals and often leads to significant distress for family members. The social economic burden caused by ASD in 2015 was 268 billion dollars and is expected to reach 461 billion by 2025 in United Sates (4). In China, ASD results in higher economic burden compared to physical disability and other mental disabilities (5). Research suggests that 76.2% of the total income of ASD families is used for the management of the disease (6). Therefore, early diagnosis and effective treatment is very important for improving the prognosis of autism and reducing the family and social burdens.

To develop more effect interventions, comprehensive assessment of symptoms of autism is needed. The Aberrant Behavior Checklist (ABC) is a caregiver rating scale designed to assess the severity of core symptoms and comorbid emotional and behavioral problems of autism. It was originally developed to measure treatment outcome in mental disability by M.G. Aman (7). It was later applied to the assessment of several diseases with obvious behavioral disturbances, especially developmental disorders (including ASD) but also genetic diseases such as Down Syndrome and Fragile X Syndrome (8, 9). ABC can be used for both children and adults. It is now a widely used tool for measuring treatment outcome of both drug and behavioral interventions of autism (10, 11). Compared to other tools used in autism, ABC describes behavior problems more comprehensively. In addition to depicting symptoms in communication and stereotypical behavior, it can also investigate aspects such as emotional stability, attention and hyperactivity, which are not always included in other commonly used scales such as the Autism Behavior Checklist and Social Response Scale. The ABC is easy to use. It can be filled out by the caregiver alone in 15 min without professional guidance. It has been translated into more than 20 languages (12). Its psychometric properties in autism have been established (13–15). The revision of the Traditional Chinese edition has also been carried out in Hong Kong with satisfactory results (16). However, language usage in Hong Kong is significantly different from the mainland because of differences in culture and context, and may even lead to ambiguity. Thus, it is necessary to introduce a Simplified Chinese edition. This study introduced and explored the reliability and validity of the Simplified Chinese version of ABC to provide a basis for the use of the scale and contribute to further research on the treatment of autism in China.

## Results

### Research Setting and Participants

This study included 799 patients who were diagnosed with autism according to both the International Classification of Diseases 10th edition and the Diagnostic and Statistical Manual of Mental Disorders 4th edition (DSM-IV) criteria upon attending child and adolescent psychiatrists or higher-level physicians at Peking University Sixth Hospital from April 2008 to November 2019. Among them, 689 were recruited from the outpatient clinic of Peking University Sixth Hospital, while 110 were recruited from a special education school in Qingdao city in Shandong province. The study was approved by the ethics committee of Peking University Sixth Hospital. All families provided informed consent before data collection.

All primary caregivers who interacted with the patient on a daily basis filled out the Simplified Chinese version of the Aberrant Behavior Checklist (SC-ABC). For criterion validity, 743 participants filled out the Autism Behavior Checklist, 319 filled out the Achenbach Child Behavior Checklist (CBCL), and 433 filled out the Attention Deficit/Hyperactivity Disorder-Rating Scale-IV. In 84 subjects, both parents who knew the patients well-filled out the SC-ABC separately to assess interrater reliability. Forty-four patients' caregivers filled out the SC-ABC again 2 weeks after the first assessment to evaluate test-retest reliability.

### Instruments

#### The Simplified Chinese Version of the Aberrant Behavior Checklist (SC-ABC)

The SC-ABC has a total of 58 items. Each item is scored 0–3 by the caregiver according to the patient's behaviors in the past 1 month. The 58 items can be divided into five factors, which are (I) irritability, (II) lethargy, (III) social withdrawal, (IV) stereotypic behavior, (V) hyperactivity, and (VI) inappropriate speech (7). The checklist was translated into Simplified Chinese by the researchers of Peking University Sixth Hospital with the consent of the developer and was proofread and back-translated by two other professors in child adolescent psychiatry from Peking University Sixth Hospital.

#### Autism Behavior Checklist

The Autism Behavior Checklist is a classic screening tool for autism developed by Krug in 1980 (17). It can reflect the core symptoms of autism and showed good classification accuracy in a previous study (18). It was introduced and translated by X.L. Yang et al. in 1993, and the Simplified Chinese version achieved good reliability and validity verification (19). The checklist has a total of 57 items and five subscales: sensory behavior, social relating, body and object use, language and communication skills, and social and adaptive skills. Each item was scored from 0 to 3. Higher scores indicate more severe symptoms. In this study, social relating, body/object use and language/communication skills subscales of this scale were used as validity criterion for lethargy, stereotype and inappropriate speech subscales of SC-ABC, respectively.

#### Achenbach Child Behavior Checklist (CBCL)

The CBCL is a widely used tool for behavior assessment. It was originally developed by T.M. Achenbach, and introduced to mainland China and translated by L.Y. Su et al. in 1996, who also established population norms and evaluated its psychometric properties (20). The checklist consists of 113 items with scores of 0–2. The 113 items can be divided into 8–9 factors according to different genders and age groups. Higher scores indicate more severe symptoms. Here, we used the aggressive behavior subscale for validity criterion evaluation of the irritability subscale of the ABC.

#### Attention Deficit/Hyperactivity Disorder-Rating Scale-IV (ADHD-RS-IV)

This is a checklist of 18 items organized according to the diagnostic criteria of attention deficit/hyperactivity disorder (ADHD) in DSM-IV. It is commonly used for ADHD screening. Each item is scored from 0 to 3. The 18 items are grouped into 2 subscales: the inattention subscale and the hyperactivity-impulsivity subscale. Higher scores indicate more severe symptoms (21). The total score of this scale was used as a validity criterion for the hyperactivity subscale of ABC.

### Statistical Analysis

We calculated means and standard deviations for the descriptive analysis of normative data. We conducted Spearman's rank correlation coefficients and intragroup correlation coefficient for subscales and total scores to assess both interrater and test-retest reliability. We measured the internal consistency of scales by Cronbach's α. We also conducted Spearman's rank correlation coefficients between subscale scores of SC-ABC and subscale scores of Autism Behavior Checklist, the aggressive behavior subscale score of CBCL, and the total score of ADHD rating scale to assess criterion validity. In addition to calculating the criterion validity of the entire sample, due to the large age span of the sample, we calculated the criterion validity in three age groups to assess whether there were age differences. We used maximum likelihood estimation for confirmatory factor analysis (CFA). We chose the original five-factor model for CFA (7) and conducted the χ2/df, Standardized Root Mean Square Residual (SMR) and Root Mean Square Error of Approximation (RMSEA) to assess model fit. We also tested factor loadings of all items. Spearman's rank correlation coefficients, intragroup correlation coefficient and Cronbach's α were performed using Statistical Package for the Social Sciences software, version 24.0; CFA was performed using AMOS 22.0. All statistical tests were two-tailed with an alpha level = 0.05.

## Materials and Methods

There were 650 males and 129 females; patients' ages ranged from 1.5 to 33 years old, with an average age of 8.9 ± 5.6 years. The normative data and SC-ABC mean total scores are shown in Table 1.

**Table 1:** Normative data of the sample.

**Age range**	**Age (SD)**	**N**		**ABC total score (SD)**
		**Male**	**Female**	
<12 years	5.9 (2.4)	463	84	54.5 (29.1)
12–18 years	14.4 (1.8)	156	30	40.6 (27.5)
>18 years	23.6 (3.8)	31	14	34.8 (23.1)
Entire sample	8.9 (5.6)	650	129	50.0 ± 29.2

### Interrater Reliability

The intragroup correlation coefficients (ICC) of the five subscales (irritability, lethargy, stereotype, hyperactivity, inappropriate speech) and the total score of SC-ABC were respectively 0.90, 0.89, 0.87, 0.90, 0.90, and 0.92 (all *P* < 0.01). The results of the Spearman's rank correlation coefficients are shown in Table 2. The correlation coefficient of the hyperactivity subscale was 0.78, while correlation coefficients of the other four scales were all over 0.80 (all *P* < 0.01).

**Table 2:** Spearman's rank correlation coefficients for reliability.

	**Interrater reliability** ***N*** **=** **84**		**Test-retest reliability** ***N*** **=** **44**	
	**ICC**	**R**	**ICC**	**r**
Lethargy	0.89**	0.80**	0.97**	0.94**
Inappropriate speech	0.90**	0.84**	0.93**	0.84**
Stereotype	0.87**	0.83**	0.97**	0.93**
Irritability	0.90**	0.83**	0.94**	0.86**
Hyperactivity	0.90**	0.78**	0.97**	0.91**
Total score	0.92**	0.85**	0.97**	0.94**

** *P < 0.01*.

### Test-Retest Reliability

The ICC of the five subscales (irritability, lethargy, stereotype, hyperactivity, inappropriate speech) and the total score of SC-ABC were 0.94, 0.97, 0.97, 0.97, 0.93, and 0.97, respectively (all *P* < 0.01). The results of the Spearman's rank correlation coefficients are shown in Table 2. All subscales and total scores had correlation coefficients over 0.80 with *P* < 0.01.

### Internal Consistency

The Cronbach's α of the five subscales (irritability, lethargy, stereotype, hyperactivity, inappropriate speech) and the total score of SC-ABC were 0.90, 0.91, 0.85, 0.91, 0.75, and 0.96, respectively (all *P* < 0.01).

### Criterion Validity

The results of the correlation analysis in the whole sample and the three age groups are shown in Tables 3–6. The scores of the lethargy, stereotype, inappropriate speech, and hyperactivity subscales of SC-ABC positively correlated with scores of their validity criterion in the entire sample and all three age subgroups. Their correlation coefficients ranged between 0.42 and 0.76 (all *P* < 0.01). The score of the irritability subscale of SC-ABC was positively correlated with aggressive behavior in CBCL for patients under 16 years old. The correlation coefficients ranged from 0.63 to 0.67 (all *P* < 0.01). However, CBCL was not applicable to participants over 16 years old, so there are no data for the group aged over 18 years old.

**Table 3:** Spearman's rank correlation coefficients for validity (entire sample).

	**SC-ABC Subscales**				
	**Lethargy**	**Inappropriate speech**	**Stereotype**	**Irritability**	**Hyperactivity**
Autism behavior checklist (*n* = 743)					
Social relating	0.57**	–	–	–	–
Body and object use	–	–	0.57**	–	–
Language and communication skills	–	0.41**	–	–	–
CBCL (*n* = 319)					
Aggressive behavior	–	–	–	0.65**	–
ADHD-RS (*n* = 433)					
Total score	–	–	–	–	0.73**

** *P < 0.01*.

**Table 4:** Spearman's rank correlation coefficients for validity (age < 12).

	**SC-ABC subscales**				
	**Lethargy**	**Inappropriate speech**	**Stereotype**	**Irritability**	**Hyperactivity**
Autism behavior checklist (*n* = 525)					
Social relating	0.53**		–	–	–
Body and object use	–	–	0.58**	–	–
Language and communication skills	–	0.39**	–	–	–
CBCL (*n* = 192)					
Aggressive behavior	–	–	–	0.66**	–
ADHD-RS (*n* = 261)					
Total score	–	–	–	–	0.69**

** *P < 0.01*.

**Table 5:** Spearman's rank correlation coefficients for validity (age 12–18).

	**SC-ABC subscales**				
	**Lethargy**	**Inappropriate speech**	**Stereotype**	**Irritability**	**Hyperactivity**
Autism behavior checklist (*n* = 174)					
Social relating	0.62**		–	–	–
Body and object use	–	–	0.55**	–	–
Language and communication skills	–	0.46**	–	–	
CBCL (*n* = 127)					
Aggressive behavior		–	–	0.63**	–
ADHD-RS (*n* = 144)					
Total score	–	–	–		0.72**

** *P < 0.01*.

**Table 6:** Spearman's rank correlation coefficients for validity (age > 18).

	**SC-ABC subscales**				
	**Lethargy**	**Inappropriate speech**	**Stereotype**	**Irritability**	**Hyperactivity**
Autism behavior checklist (*n* = 44)					
Social relating	0.67**	–		–	
Body and object use	–	–	0.42**	–	
Language and communication skills	–	0.51**	–	–	
CBCL (*n* = 0)					
Aggressive behavior	–	–	–	–	–
ADHD-RS (*n* = 28)					
Total score	–	–	–		0.76**

** *P < 0.01*.

### Structural Validity

The five-factor model according to the original version of ABC was used in the analysis. The model fit indices were as follows: χ2/df = 3.105, SMR = 0.062, and RMSEA = 0.052. Standard factor loadings ranged from 0.312 to 0.785, as shown in Table 7.

**Table 7:** Standard factor loading of the SC-ABC.

**Irritability**		**Lethargy**		**Stereotype**		**Hyperactivity**		**Inappropriate speech**	
**Item**	**Loading**	**Item**	**Loading**	**Item**	**Loading**	**Item**	**Loading**	**Item**	**Loading**
2	0.409	3	0.387	6	0.77	1	0.599	9	0.435
4	0.396	5	0.663	11	0.694	7	0.779	22	0.587
8	0.688	12	0.388	17	0.785	13	0.635	33	0.757
10	0.574	16	0.636	27	0.547	15	0.676	46	0.756
14	0.626	20	0.636	35	0.736	18	0.769		
19	0.722	23	0.486	45	0.658	21	0.599		
25	0.312	26	0.506	49	0.541	24	0.75		
29	0.718	30	0.646			28	0.702		
34	0.51	32	0.407			31	0.514		
36	0.625	37	0.712			38	0.672		
41	0.661	40	0.755			39	0.675		
47	0.615	42	0.606			44	0.457		
50	0.400	43	0.682			48	0.685		
52	0.407	53	0.482			51	0.642		
57	0.677	55	0.678			54	0.465		
		58	0.752			56	0.655		

## Discussion

### Reliability

The ICC for test-retest reliability of the five subscales and the total scores of SC-ABC were ~0.90. The results are similar to the median ICC of 0.94 that Siegfrid reported in a Traditional Chinese version with a 4 week retest gap (16). The Spearman's rank correlation coefficients for the five subscales and total scores of SC-ABC were all above 0.85, which was greater than those reported in a previous study with retest gap of 4 weeks by M.G. Aman, and similar to another result reported by Miller with a retest gap of 2 weeks (22, 23). Cohen proposed criteria in 1992 stating that a correlation is negligible when r is below 0.1, small when r ranges from 0.10 to 0.30, moderate when r ranges from 0.30 to 0.50, and large when r is >0.50 (24). According to these criteria, the result reflects good test-retest reliability.

The results also indicate good interrater reliability in the SC-ABC. The subscales and total scores all had ICC ranging from 0.87 to 0.92, which were more favorable compared to a previous study of the Traditional Chinese version (16). Spearman's correlation coefficients ranged from 0.78 to 0.85, which is greater than in the report of the Japanese version (25).

The Cronbach's alpha in previous studies of the psychometric properties of ABC in autism populations ranged from 0.73 to 0.94, which is comparable to the Cronbach's α ranging from 0.75 to 0.96 in our analysis. Similar to the previous findings, we found the lowest, but acceptable, Cronbach's α in the inappropriate speech subscale (14, 15, 26).

### Validity

The lethargy, inappropriate speech and stereotype subscales of SC-ABC showed moderate to large correlation with their validity criterion in the entire sample, as well as in each of the three age groups, which indicate that the three subscales have good validity for assessment of core symptoms of autism. Our results also show that the correlation coefficients between lethargy, inappropriate speech subscales and their validity criteria increase by age (for lethargy 0.53–0.67; for inappropriate speech 0.39–0.51). This may be due to social interaction and communication becoming significant with social demand increase when individuals grow up (27). Therefore, some deficits of social interaction and communication were not considered to be problems by some parents when their children were young, which may lead to inconsistent results for the same aspect in different assessment tools. When patients grow up, their deficits of social interaction and communication get more attention from parents, which leads to more objective and consistent assessment, resulting in the validity increase. In our study, the correlation coefficients between the stereotype subscale and its validity criterion decreased with age; they were large in groups under 18 years old and moderate in groups over 18 years old. This may be because the detailed text of the two subscales are not totally the same, and symptoms change with age. According to the text of the two checklists, seven items of the stereotype subscale of SC-ABC are about repetitive behaviors related to the body, such as shaking the head, body and limbs, which may be less impairing for the patients and their family. As a result, parents likely do not pay a great deal of attention to these behaviors, and their scores tend to remain unchanged over time. In contrast, half of the 12 items in the criterion body/object use subscale of the Autism Behavior Checklist are about self-injury, destruction of objects and complex ritual behaviors that could lead to significant impairment of social function. These behaviors are more noticeable and tend to be corrected, so that the subscale score decreases with age. This may be a potential reason for the decreased correlation coefficient between stereotype and body/object use. In general, the three subscales of the SC-ABC show reasonable criterion validity, suggesting that it could be used to assess the core features of autism.

We used total score of ADHD-RS as the criterion for the hyperactivity subscale of SC-ABC and achieved good validity. Aggressive behavior and irritability are related, but not completely equivalent. However, with no more suitable tool, we chose the aggressive behavior subscale of CBCL as the criterion for the irritability subscale of SC-ABC and achieved a large correlation between the two scales. Both hyperactivity and irritability are common and disturbing in patients with autism. These behaviors often significantly impair the patients' social function and have great impact on their family but can be effectively improved by behavior intervention and medications. The SC-ABC has good criterion validity in these two subscales and can be further used for evaluation of hyperactivity and irritability in patients with ASD. However, it must be noted that there are no CBCL data in adult participants, and therefore, the criterion validity of the irritability subscale in adult populations still need to be further explored.

This study also explores the structural validity of SC-ABC by CFA. We fitted the five-factor model proposed by the original author M.G. Aman. Since the checklist was developed, there have also been four factor and six factor models proposed by other researchers (9, 13). M.G. Aman compared these models in autism populations and reported that the original five factor model still has the best fit (14). Therefore, we used the five-factor model for CFA in this study. The results showed that χ2/df was between 3 and 5, and RMSEA and SMR were in the range of 0.05–0.08. According to the guideline by Browne and Cudeck, an RMSEA below 0.05 suggests a good model fit, between 0.05 and 0.08 suggests a reasonable fit, between 0.08 and 0.10 suggests a marginal fit, and above 0.10 suggests an unacceptable fit (28). Our findings indicate a reasonable model fit. For SMR, a value of <0.08 indicates a good model fit (29). Fifty-five of the factor loadings were >0.4, while 48 items had a factor loading of more than 0.5, comparable to previous reports. Four items had a factor loading below 0.4. Item 25 “depressed mood” in subscale irritability with factor loading 0.312 was relatively low, as a previous study reported (14). Indeed, it is understandable that patients with depressed mood are not always irritable. It may be a structural issue of the original model that should be optimized. The remaining 3 items did not perform poorly in the previous studies. We checked the original text and translation and confirmed that there were no translation errors. The low factor loading may be related to cultural compatibility. Considering the overall results, the model fit of the Simplified Chinese version is acceptable.

### Limitations

Although participants in this study came from all over the country, the proportion of rural participants was relatively small. The sampling method may introduce a certain amount of bias. The patient population should be expanded to include more patients from rural communities for further verification if possible. We were also unable to acquire “gold standard” criterion data such as Autism Diagnostic Observation Schedule or Autism Diagnostic Interview—Revised. Furthermore, this study has not been able to incorporate the criteria for the irritability subscale in patients aged over 18 years old. If appropriate tools are available, further exploration of the criterion validity of the irritability subscale should be conducted, especially in an adult sample.

## Conclusions

In conclusion, the Simplified Chinese version of the ABC has good reliability and validity in Chinese ASD patient populations, while the criterion validity of the irritability subscale in adults still needs to be further examined.

## Data Availability Statement

The datasets generated in this article are not readily available because approval of the ethic committee of Peking University Sixth Hospital is needed. The datasets are available from the corresponding author on reasonable request with the permission of the Ethic Committee. Requests to access the datasets should be directed to @bjmu.edu.cn.

## Ethics Statement

The studies involving human participants were reviewed and approved by the Ethic Committee of Peking University Sixth Hospital. Written informed consent to participate in this study was provided by the participants' legal guardian/next of kin. Written informed consent was obtained from the individual(s), and minor(s)' legal guardian/next of kin, for the publication of any potentially identifiable images or data included in this article.

## Author Contributions

JL, XL, and SK made contributions to the conception and design of the study. YG, JL, and JM made substantial contributions to translation and proofreading of SC-ABC. All the authors were responsible for data acquisition. SK and JM were responsible for data analysis. SK wrote the first draft of the manuscript and JL, XL, and LX critically reviewed and revised the initial draft. All authors have approved the final version of the submitted manuscript.

## Conflict of Interest

The authors declare that the research was conducted in the absence of any commercial or financial relationships that could be construed as a potential conflict of interest.

## Abbreviations

ADHD: Attention Deficit Hyperactivity Disorder
ADHD-RS: Attention Deficit Hyperactivity Disorder Rating Scale
CBCL: Child Behavior Checklist
CFA: confirmatory factor analysis
DSM-IV: Statistical Manual of Mental Disorders 4th edition
ICD-10: Classification of Diseases 10th edition
SC-ABC: Simplified Chinese Aberrant Behavior Checklist
SMR: Standardized Root Mean Square Residual
RMSEA: Root Mean Square Error of Approximation.
